# Supplementary material for: Rural–Urban Disparities in Patient Care Experiences among Prostate Cancer Survivors: A SEER-CAHPS Study
Source: Cancers (Basel). 2023 Mar 23;15(7):1939. doi: 10.3390/cancers15071939 (PMC10093298; doi:10.3390/cancers15071939)
Supplement: Supplementary file 1 [file cancers-15-01939-s001.zip › cancers-2269924-supplementary.pdf]

Supplementary Table S1: Least-square mean estimates and adjusted differences in mean patient experience measures by rural-urban status

Patient Care Experience Measure	Big Metro	Metro			Rural		
	Least-square mean estimate ± SE	Least-square mean estimate ± SE	Adjusted difference from Big Metro (95% CI)	p-value	Least-square mean estimate ± SE	Adjusted difference from Big Metro (95% CI)	p-value
**Getting needed care (n=2,460)**	84.5 ± 6.2	85.0 ± 6.2	0.5 (-1.2 to 2.2)	0.552	86.2 ± 6.3	1.6 (-1.1 to 4.4)	0.238
**Getting care quickly (n=2,864)**	59.7 ± 6.0	60.7 ± 6.0	0.9 (-1.0 to 2.9)	0.357	60.6 ± 6.1	0.9 (-2.3 to 4.0)	0.599
**Doctor communication (n=2,504)**	85.2 ± 4.9	85.5 ± 4.9	0.3 (-1.0 to 1.6)	0.673	86.9 ± 5.0	1.7 (-0.4 to 3.8)	0.119
**Getting needed prescription drugs (n=1,811)**	91.4 ± 6.2	92.9 ± 6.2	1.4 (-0.5 to 3.4)	0.153	94.2 ± 6.3	2.8 (-0.5 to 6.0)	0.097
**Customer service (n=878)**	68.5 ± 8.7	67.0 ± 8.7	-1.5 (-5.2 to 2.2)	0.429	65.2 ± 9.2	-3.2 (-9.8 to 3.4)	0.342
**Primary care provider rating (n=2,494)**	87.3 ± 4.4	87.7 ± 4.4	0.4 (-0.7 to 1.6)	0.469	87.7 ± 4.5	0.4 (-1.5 to 2.3)	0.645
**Specialist rating (n=2,089)**	86.2 ± 5.0	86.4 ± 5.1	0.2 (-1.1 to 1.5)	0.767	85.9 ± 5.2	-0.3 (-2.5 to 1.9)	0.780
**Health plan rating (n=2,956)**	85.2 ± 4.5	84.2 ± 4.5	-1.0 (-2.4 to 0.4)	0.151	83.9 ± 4.6	-1.4 (-3.7 to 0.9)	0.238
**Overall care rating (n=2,913)**	87.8 ± 4.1	87.8 ± 4.0	0.0 (-1.2 to 1.3)	0.953	87.8 ± 4.1	0.0 (-2.1 to 2.0)	0.981

SE – Standard Error

CI – Confidence Interval
